# Development of a Scoring System to Assess Feather Damage in Canadian Laying Hen Flocks

**DOI:** 10.3390/ani9070436

**Published:** 2019-07-10

**Authors:** Caitlin Decina, Olaf Berke, Nienke van Staaveren, Christine F. Baes, Alexandra Harlander-Matauscheck

**Affiliations:** 1 Department of Population Medicine, Ontario Veterinary College, University of Guelph, Guelph, ON N1G 2W1, Canada; 2 Department of Animal Biosciences, Ontario Agricultural College, University of Guelph, Guelph, ON N1G 2W1, Canada

**Keywords:** feather damage, scoring, visual, reliability, poultry

## Abstract

**Simple Summary:**

Feather damage is a continuous welfare challenge in the management of egg-laying hens. Canada is currently transitioning from conventional cages to alternative housing systems, where the risk of feather damage may increase due to larger group sizes. This change increases the need for continued and reliable assessment of flock feather condition, for which Canada does not have a standard method. Within this study, a feather damage scoring system and visual scoring guide were developed, with the ultimate goal of streamlining and increasing plumage assessment of laying flocks by farmers on Canadian commercial farms. Two differing feather scoring systems (LayWel and AssureWel) were compared based on user-friendliness and reliability. The AssureWel scoring system was easiest to use and achieved the most consistent outcomes among scorers for the back area of the body. This informed the design of a modified version with scoring levels from 0 to 2 for a sample of 50 birds per flock, along with an informational, visual guide for farmers. Training of farmers to use this simplified scoring system under commercial conditions can provide a benchmarking tool for feather damage levels, as well as a way to measure the success of management strategies to prevent and control feather damage.

**Abstract:**

Feather damage (FD) due to feather pecking behavior is an ongoing welfare concern among commercial egg-laying hens. Canada’s current transition from conventional cage housing to alternative housing systems, where FD can spread easily within large flocks, underlines the need for frequent and accurate assessment of plumage condition. A standardized methodology for assessing FD in Canada does not yet exist. To improve FD assessment on commercial farms, a FD scoring system and visual scoring guide for farmers were developed. Two existing plumage scoring systems, LayWel and AssureWel, which differ in level of detail and bird handling, were assessed for ease of use, and intra- and inter-observer reliability. Practical application of the AssureWel scoring system was greatest, with strong intra- and inter-observer reliability for the back region of the body (weighted kappa = 0.88 for both measures) in small-scale flocks. This informed the creation of a modified version of the AssureWel system, which included three scoring levels and the visual assessment of 50 birds per flock. An accompanying guide was developed including sampling instructions and depictions of the scoring scheme, both written and visual. This simplified scoring system can serve as a benchmarking tool for FD prevalence, and can allow for future effectiveness assessments of management strategies to prevent and control FD; however, farmers should be trained to apply this system under commercial conditions.

## 1. Introduction

Feather pecking (FP) is a serious and learned behavioral problem in laying hen flocks [1], which leads to feather damage (FD), including feather loss, and, in more severe cases, to cannibalism and great economic loss to the farmer [2]. An intact feather cover serves many functions, of which the most noticeable is enabling a bird to move, fly, and navigate its environment [3], which is especially important in non-cage housing systems [4]. Additionally, it is important in thermoregulation and protection of the skin [5,6]. A non-intact feather cover is a multifactorial problem with potentially different underlying etiologies and associated risk factors [7,8]. The problem farmers face is being aware of, and determining the extent of, FD in commercial flocks of laying hens to identify and treat feather cover issues early on. It is, therefore, important to assess and monitor the presence and severity of FD as a proxy for FP, as direct observation of FP behavior can be difficult and time-consuming to observe in commercial settings. Continuous FD assessment assumes an easy to handle and accurate scoring tool available to farmers. 

Numeric rating scales for FD scoring schemes were developed and employed in past studies on the topic of FP and FD. Current scoring methods differ in the amount of detail they record with certain scoring systems using comprehensive 4–6-point scales [5,9,10]. Detailed scoring scales allow the capture of more comprehensive information regarding severity or extent of damage, rather than simple presence or absence. These scoring systems range from including information on intact feather cover or no damage, to the presence of ruffled feathers or few feathers missing, to substantial damage, such as bald patches indicated in size by centimeters in diameter or percentage of the considered body area affected [9,10]. In contrast, other schemes, such as those used in the welfare assessment protocols of Welfare Quality^®^ [11] and AssureWel [12], use more condensed three-point scales ranging from scores of 0 to 2 indicating no damage, moderate wear or small bald patches (<5 cm), and large bald patches (>5 cm), respectively. Some frequently used schemes such as the LayWel system [5] do not provide descriptive definitions of their scores, but rather provide photographs as a reference (1–4 scoring scale), while the AssureWel system provides both definitions of scores (0–2 scoring scale) and photographs [12]. 

FD scoring methodology also differs in the type of feathers to be assessed (i.e., body or flight feathers [9,10]), the number of body areas to be assessed (ranging from 3–11 areas [9,10,11,12]), and whether or not birds are captured and handled during the assessment. Bright et al. [10] and Kjaer et al. [13] showed that a visual assessment of laying hens gave results similar to assessments where birds were captured and handled, and visual assessment was considered less stressful for the birds. 

In general, systems with more scoring categories and a high number of body areas to be scored are more time-intensive, and achieving reliability between observers is more difficult [12,14] than when using simplified systems with few categories and body areas for assessment. While simplified systems can, therefore, be more easily implemented commercially, categories that still accurately reflect FD severity and target known body areas affected by FD are important for valid assessment. 

Currently, there is no standardized program applied on Canadian egg farms to consistently record the prevalence of FD, although some farms might apply their own scoring systems as part of their animal care program. Due to the fact that Canada committed to banning conventional cages and is transitioning to all furnished cages and non-cage housing systems [15], where intact feather cover will be key to bird welfare, farmers’ awareness of feather cover care and early identification and treatment of feather cover issues will be crucial. For these reasons, farmer-friendly monitoring tools that are feasible, e.g., where farmers can assess a subset of birds to represent an entire flock, are needed to assess FD under commercial conditions.

The goal of this study was to develop an accurate, reliable, and feasible FD scoring system that could ultimately be applied on commercial farms to have a nation-wide, standardized methodology for assessing feather cover in laying hens. Specific objectives were to (i) evaluate the reliability of two existing scoring systems, using a detailed system (LayWel) versus a simplified alternative (AssureWel), (ii) assess these two systems’ timeliness and ease, and (iii) adjust their attributes to suit the Canadian commercial setting.

## 2. Materials and Methods 

Reliability testing of FD scoring systems was conducted on laying hens involved in current FP research on the University of Guelph campus. In the current study, FD was defined as both the destruction of feathers and their loss. A total of 117 non-beak-trimmed white Leghorn hens (approximately 64 weeks of age) were housed in two rooms with floor pens (118 length (L) × 118 width (W) × 365 height (H) cm) in groups of approximately nine birds/pen. Each bird was individually identifiable via numbered silicone backpacks (approximately 6 × 8 cm). The sample size used was 50 birds per flock as advised by the AssureWel system, used previously in FP-related studies such as those by de Haas et al. [16] and Heerkens et al. [17]. Main et al. [12] also noted that a sample of 50 birds was the maximum number of birds that could be reasonably assessed considering time constraints. 

### 2.1. Intra-Observer Agreement 

A naïve member of the research team (without previous experience with the scoring system) scored for FD from outside the pens per the LayWel and AssureWel systems (Table 1), and this was repeated 1–2 days later to estimate intra-observer reliability. Scoring was done from outside the pens in one room (approximately 50 birds) to mimic commercial conditions where producers would be asked to visually inspect their birds to increase the feasibility of the methodology. As such, only body areas visible without handling the birds were assessed (i.e., neck, back, wings, and tail) while body areas that were not visible were excluded (i.e., vent and breast). The back area covered by the silicone backpack was excluded; only the visible area of the lower back was assessed. Additionally, an overall score was assigned based on the general appearance of the entire bird. Scoring system training consisted of familiarization with the various scoring scales and required body areas through the reading of their respective instructions and attributes beforehand. Each system’s instructions were also on hand during scoring for reference. Following this, the same observer completed a second session of FD assessment using only the AssureWel system, after initial intra-observer testing of the scoring system showed the most promise for the AssureWel system, to further assess the intra-observer reliability after previous experience. 

### 2.2. Inter-Observer Agreement 

A second naïve observer was included in further sessions to estimate inter-observer reliability using each scoring system (Table 1), again using only easily visible body areas consistent with those scored during the intra-observer sessions. The inter-observer reliability assessment coincided with FD assessment performed for a separate, unrelated experiment. Due to the nature of this experiment, a larger sample of 117 birds (the entire study population of birds housed in the floor pens) was captured and handled to assess FD using the LayWel scoring system. For inter-observer reliability estimation when using the AssureWel system, we used the original method of visual assessment only with the established sample of 50 birds. Additionally, during the visual assessment with the AssureWel system, observers recorded the presence or absence of a naked patch of skin on the body of the bird as a singular scoring feature to see if scorers could agree on an absence of feathers, even if the exposed skin area was very small. Both observers scored the same birds under the exact same conditions. 

### 2.3. Statistical Analysis

Weighted kappa (WK) values were calculated to correct for agreement by chance while taking into consideration the ordinal nature of the data, thereby also accounting for the extent of the disagreement between results [18]. Kappa values were interpreted following the suggested classifications put forth by Landis and Koch [19] and presented in Petrie and Watson [20] where *κ* ≤ 0.20 is “poor”, 0.21 ≤ *κ* ≤ 0.40 is “fair”, 0.41 ≤ *κ* ≤ 0.60 is “moderate”, 0.61 ≤ *κ* ≤ 0.80 is “substantial”, and *κ* > 0.80 is “good”. Additionally, Spearman rank correlations were calculated between the overall score and the scores given to the individual body areas, and between the scores given by the two observers. All agreement calculations were performed using SAS^®^ statistical software, version 9.4 (SAS Institute Inc., Cary, NC, USA [21]). 

### 2.4. Scoring System Modifications 

The reliability results and time to completion of each assessed scoring system informed a modified scoring system. Following this, supplementary documents were constructed to aid farmers in the on-farm use of the system. A FD scoring guide was produced, which includes photographic references for each score for both white- and brown-feathered birds. The guide presents all features of the scoring system and the sample size (50 birds) required from each flock. Further instructions include schematics on how best to sample birds randomly from all areas of the barn, similarly to those recommended by the AssureWel system [12] and Welfare Quality project [11]. Additionally, scoring sheets were created showing photographic scales and a real scale size reference for featherless areas, which allowed for organized recording of scores for both white and brown-feathered birds.

The final system and supplementary materials were reviewed by staff at the University of Guelph’s Arkell Poultry Research Station, as well as nearby farms representing the different housing systems, to ensure clarity of instructions and feasibility of the procedure. Three Arkell staff members were given a scoring sheet and the scoring guide to assist with hen scoring within a small flock housed in furnished cages. Each member randomly selected 50 different white hens from cages in different tiers and different locations within the room. Their time to completion was recorded to estimate how long the scoring procedure might take on a commercial farm. A short discussion followed with the staff regarding the ease of the scoring system and their thoughts on its application under probable commercial conditions. Each staff member’s scores were evaluated to determine how their estimated FD prevalence compared to each other.

## 3. Results

### 3.1. Intra-Observer Agreement

Results of the first intra-observer reliability session are presented in Table 2. Weighted kappa (WK) values for the LayWel scoring system were moderate to substantial, with the lowest reliability for the neck region. The AssureWel system showed moderate to good WK depending on the body region, with the back showing the greatest reliability among the individual body regions. During reliability testing, the AssureWel system took approximately 30 min per 50 birds to complete, while the LayWel system was more time-intensive at approximately 50 min per 50 birds. 

The second session of intra-observer reliability testing was conducted with the AssureWel scoring system only after AssureWel showed more promising WK values in the first session. These results showed considerable improvement for neck and back regions, as well as for the overall score. Wing and tail regions continued to show weaker kappa values (Table 3). The score for the back region was most strongly correlated with the overall score (*r* = 0.88).

### 3.2. Inter-Observer Agreement

The agreement figures for inter-observer reliability between the two researchers are presented in Table 4. This initial assessment showed that, for the neck, good WK values were achieved using the LayWel system; however, for the back, no WK could be calculated due to the fact that one observer used more score categories than the other. With the AssureWel scoring system, both the neck and back areas showed substantial to good WK values. Similarly, scores of the back region from both observers were highly correlated (*r* = 0.90). In contrast, lower levels of agreement were found for the wing and tail areas between observers.

Additionally, observers recorded the presence or absence of a naked patch of skin as a singular scoring feature to see if scorers could agree on an absence of feathers, even if the exposed skin area was very small. At this stage, no size limitation was placed on the naked area. This scoring feature showed a strong kappa value (κ = 0.84 ± 0.08) based on the assessment of 50 birds.

### 3.3. Final Feather Damage Scoring System

**Figure 1:**
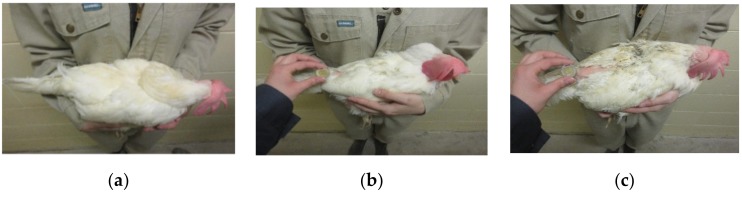

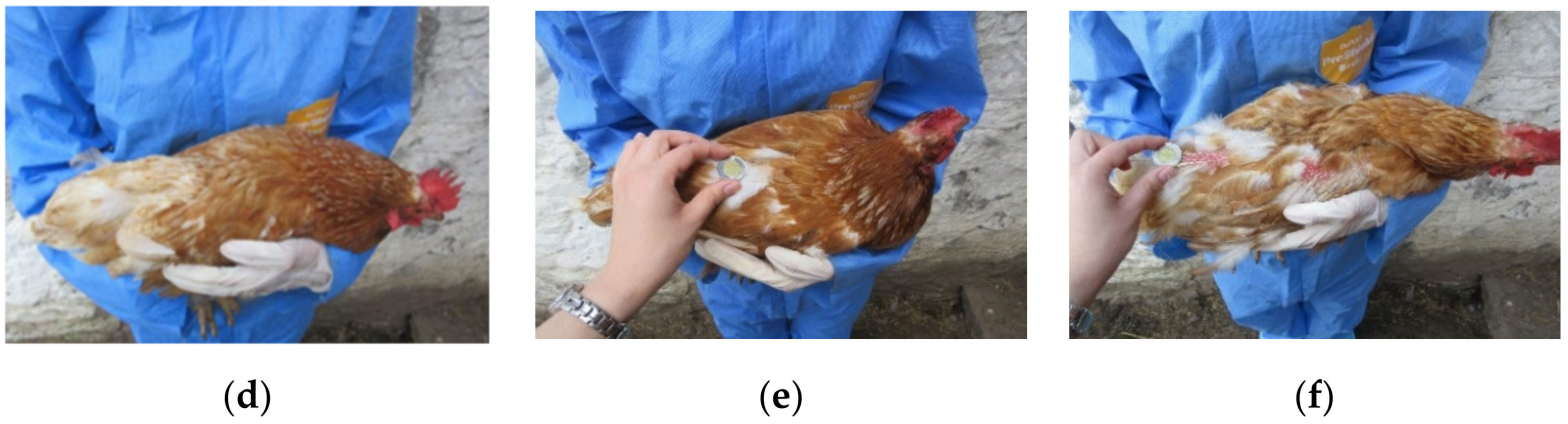
Visual representation of the developed scoring system for both white- and brown-feathered birds: score of 0 (**a**,**d**); score of 1 (**b**,**e**); score of 2 (**c**,**f**).

After assessment of both the more detailed LayWel system and the simplified AssureWel system, the final scoring system decided upon by the research team was a modification of the AssureWel scheme, presented in Table 5 and Figure 1. The three scoring categories and their general descriptions were retained, as well as the sample size of 50 birds per flock. The body areas to be scored were limited to just the back/rump region, and the size indicator of featherless skin for scores 1 and 2 was changed to that of a Canadian two-dollar coin for intuitive interpretation by Canadian farm staff. 

### 3.4. Testing in Arkell

On average, the Arkell farm staff took approximately 15 min to complete the FD scoring protocol for 50 birds. Overall, Arkell staff’s scores compared fairly well to each other in terms of percentage of the flock classified as score 0, 1, and 2 among raters. For scoring of back FD, on average, 94.7% of the flock was given a score of 0 and 5.3% of the flock was given a score of 1, while no birds with a score of 2 were observed. A maximum difference of 12% in the prevalence of FD scores was observed between raters. 

## 4. Discussion

Assessment of the existing plumage scoring systems, LayWel and AssureWel, was used to modify and refine the AssureWel scoring system into a version that could be applicable in Canada. Neither of the researchers had prior experience with assessing FD and, thus, would be more representative of the general farmer who would be scoring the birds. Due to the nationwide data collection required as part of a larger project the scoring system would be implemented in [22], the research team would not be able to collect this scoring data in person due to travel constraints. As farmers would not be trained beforehand, it was important that the scoring system was understandable and accurately applicable during first-time use, and feasibility of the methodology was crucial. On a personal level, the researchers found the AssureWel system easier and faster to use than the illustrated four-point scale of the LayWel system, and it was anticipated this would be the same for farmers. Previous studies showed that, with more detailed scoring scales, there is more room for disagreement and more training is required compared to simpler scoring systems, leading to the recommendation of using a scoring system with fewer categories [14].

Using a three-point scale allowed for the most concise representation of FD condition, as it provided a score that reflected an unaffected bird with intact feather cover, as well as both an intermediate score for birds with moderate damage, and a score for birds severely affected with a prominent bald patch. The back/rump area was selected as the only body region for scoring as it consistently showed the highest level of agreement among the regions tested in this study, as well as high levels of agreement in others [10,13]. Furthermore, it was strongly correlated with the overall score, and it is an area typically most targeted by FP behavior [7,23,24], and where FD and naked patches are least likely to be caused by abrasion from the housing system [9]. Additionally, it is an area that is most visible when visually inspecting birds. Previous observational studies used the back as a key area for FD observation as well [25,26]. Other body areas, such as the wings and tail, were not incorporated in the final scoring system because scoring of these areas was inconsistent, i.e., they showed low levels of agreement between raters, even with the simplified scales of the AssureWel system. Limiting assessment to the back region allows for a swift and reliable scoring procedure and FD assessment.

The use of a binary scale to measure the presence or absence of a naked patch to further increase feasibility and reliability was also assessed. Inter-observer reliability for this singular measure was high, but it was feared that it would lead to a loss of information regarding severity and extent of FD in flocks that could be captured with a three-point scoring scale, especially considering that farmers typically tend to score lower than researchers [14]. 

With respect to the literature, an inter-observer WK of 0.88 was reached for the back area between researchers, similar to values reported by Kjaer et al. [13], who found a WK of 0.82 between researcher teams using a visual assessment of plumage damage. Between farmers, lower values were reported (kappa of 0.50) when ranking photographs of FD damage only [27]. Some variation between assessors’ perceptions as to what is considered an acceptable level of FD is also unavoidable [12,27]; therefore, written descriptions and visual depictions of each score were included in the final system. In a trial of the modified system with the accompanying scoring guide, the three Arkell staff members who scored 50 birds within the same room containing approximately 700 birds (and, thus, likely scored different birds) found a fairly similar FD prevalence suggesting that it gave a reasonable representation of all birds within the room. Staff members also found the system easy to use, with clear straightforward instructions provided by the scoring guide. 

Intra-observer reliability of FD scoring with the AssureWel system increased after multiple sessions. It should be noted that this reflects a slight learning effect on the part of the researchers in that, with more practice and experience, reliability among raters can improve [14]. In addition to fewer scoring categories and body areas, this effect may also have played a part in the shorter duration to complete the assessment with the AssureWel system. Unfortunately, providing farmer training and assessing reliability of farmer FD scoring was outside the scope of this project, and this limitation should be acknowledged. However, farmers are free to use the visual aids and instruction material, which could increase their ability to accurately score FD within their flocks. Furthermore, the modified system employs methods very similar to the AssureWel measurement already in use for FD assessment on farms in the standards of the Royal Society for the Prevention of Cruelty to Animals (RSPCA) and Soil Association in the United Kingdom (UK) [12]. As such, this scoring system is likely a valuable tool for benchmarking FD prevalence, especially with consistent use over time as farmers get more familiar with the system. Monitoring of FD prevalence can increase the chances of early detection of FD within flocks.

Further considerations to increase the reliability of farmer collected FD scores were made to tailor the scoring system to Canadian farmers and provide a clear size indicator for the damaged area. As mentioned, in the assessment of inter-rater reliability for the presence of a naked patch, initially no size indicator was set. Most existing scoring systems define this in terms of centimeters or percentage of body area affected, which can be difficult to estimate in commercial settings, and/or without practice. Therefore, the naked patch size stipulation was modified to that of a Canadian two-dollar coin, or “Toonie”, rather than a specific linear measurement, to provide farmers with a size reference that could be easily visualized. The Toonie is unique to Canada, is something that most farmers are familiar with in terms of size, and is something that could be easily carried with them for help during scoring. A naked patch of that size, especially on the back region where damage to this area is almost exclusively caused by FP activity [9], would also be a sufficient indicator of this behavior in a flock. 

Lastly, it is important to mention two further limitations. The first is that reliability was only assessed using white-feathered birds due to the lack of a brown-feathered study population in proximity to the researchers. Therefore, we were unfortunately unable to properly assess the reliability of the scoring system when applied to brown-feathered birds. First experiences by commercial farmers (who tested out the modified FD scoring system in large flocks of both white- and brown-feathered birds) did not indicate that feather-color-specific approaches are necessary.

The second limitation relates to the small-scale housing setting (floor pens and small furnished cages) in which reliability testing was performed. These small-scale settings are not typical for commercial use; therefore, reliability estimates may vary among housing systems and differ in practice from the results found here. As such, results should not be extrapolated. Commercial farmers who tested out the FD scoring system in large flocks in both furnished cage and non-cage housing systems indicated that the time spent to complete the assessment may be longer than reported here, although it was considered feasible. Further assessment of the reliability of the FD scoring system under commercial conditions, as well as training of farmers on the scoring system, is needed to gain the most potential out of the scoring system before large-scale implementation. 

## 5. Conclusions

This study provides a rationale for the development of a feather damage (FD) scoring system, which can ultimately be applied on commercial farms where birds are housed in furnished cage and non-cage housing systems, i.e., in Canada. This scoring system, modified from the existing AssureWel protocol, was found to be reliable (weighted kappa = 0.88 for intra- and inter-observer reliability) in small-scale settings, can be easily used by farmers to assess and monitor FD within their flock, and ultimately allows for benchmarking of FD prevalence. With proper training of farmers, this scoring tool can be essential for future effectiveness assessments of management strategies to prevent and control FD.

## Figures and Tables

**Table 1:** Procedures for assessment of intra- and inter-observer reliability of feather damage (FD) scoring in laying hens based on two existing scoring systems using numeric scales (LayWel, 1–4 scale and AssureWel, 0–2 scale).

Scoring System	Reliability Test	Observer	No. of Birds
AssureWel	Intra-observer (2×) ^1^	A	54
	Inter-observer	A + B	54
LayWel	Intra-observer	A	54
	Inter-observer ^2^	A + B	117

^1^ Intra-observer reliability was estimated twice with the AssureWel scoring system to evaluate if reliability increased after previous experience. ^2^ Inter-observer reliability was estimated on a larger sample of birds with the LayWel scoring system as part of another experiment.

**Table 2:** Intra-observer reliability of feather damage (FD) scoring in laying hens based on two existing scoring systems using numeric scales (LayWel, 1–4 scale and AssureWel, 0–2 scale). Different body areas were assessed, and one overall score was given based on the overall impression of the hen. Reliability was assessed as weighted kappa (WK). Not applicable (NA) indicates cases where WK could not be calculated due to the fact that not all the same score categories were used.

	LayWel			AssureWel		
Body Area	*N* ^1^	Weighted Kappa (WK)	Standard Error	*N* ^1^	Weighted Kappa (WK)	Standard Error
Neck	53	0.49	0.232	52	0.55	0.240
Back	53	0.72	0.094	53	0.81	0.063
Wings	52	NA		53	NA	
Tail	51	0.74	0.062	48	0.68	0.096
Overall	53	NA		53	0.82	0.061

^1^ Note that the number of birds (*N*) can be slightly different due to missing values, e.g., if a certain body area of a bird was not visible and, therefore, could not be assessed by the observer.

**Table 3:** Intra-observer reliability of feather damage (FD) scoring in laying hens based on the AssureWel scoring system (0–2 scale) following initial testing. Different body areas were assessed, and one overall score was given based on the overall impression of the hen. Reliability was assessed as weighted kappa (WK).

AssureWel			
Body Area	*N*	Weighted Kappa (WK)	Standard Error
Neck	54	0.67	0.162
Back	54	0.88	0.041
Wings	54	0.36	0.199
Tail	54	0.61	0.094
Overall	54	0.85	0.059

**Table 4:** Inter-observer reliability of feather damage (FD) scoring of different body areas in laying hens based on two existing scoring systems using numeric scales (LayWel, 1–4 scale and AssureWel, 0–2 scale) between two principal members of the research team. Reliability was assessed as weighted kappa (WK). Not applicable (NA) indicates cases where WK could not be calculated due to the fact that not all the same score categories were used.

	LayWel			AssureWel		
Body Area	*N* ^1^	Weighted Kappa (WK)	Standard Error	*N* ^1^	Weighted Kappa (WK)	Standard Error
Neck	117	0.82	0.061	49	0.72	0.128
Back	117	NA		48	0.88	0.0401
Wings	117	0.58	0.058	49	NA	
Tail	116	0.74	0.034	48	0.68	0.069

^1^ Note that the number of birds (*N*) can be slightly different due to missing values, e.g., if a certain body area of a bird was not visible and, therefore, could not be assessed by one of the observers.

**Table 5:** The scoring system used by farmers on site to evaluate the feather condition and amount of feather damage present in their flock. Body areas scored were limited to the back/rump on a sample of 50 birds per flock.

Score	Body Condition
0	Intact feather cover, no or slight wear, only single feathers missing
1	Damaged feathers (worn/deformed) or bald patch visible smaller than a $2 coin
2	At least one bald patch visible greater than a $2 coin
